# Evaluation of Dual-Near-Infrared-Wavelength Photobiomodulation Therapy for the Treatment of Canine Otitis Externa: A Prospective Multicenter Pilot Study

**DOI:** 10.3390/ani16152372

**Published:** 2026-08-03

**Authors:** Federica Gesuete, Luca Luciani, Silvia Crotti, Paola Papa, Sara Muñoz Declara, Barbara Contiero, Eric Zini, Davide Travalin, Sara Spina, Giordana Zanna

**Affiliations:** 1 AniCura Istituto Veterinario di Novara, 28060 Granozzo con Monticello, NO, Italy; sara.munoz@anicura.it (S.M.D.); eric.zini@anicura.it (E.Z.); giordana.zanna@anicura.it (G.Z.); 2 AniCura Portoni Rossi Veterinary Hospital, 40069 Zola Predosa, BO, Italy; lucaluvet@yahoo.it; 3 Istituto Zooprofilattico Sperimentale dell’Umbria e delle Marche “Togo Rosati”, 06126 Perugia, PG, Italy; s.crotti@izsum.it (S.C.); p.papa@izsum.it (P.P.); s.spina@izsum.it (S.S.); 4 Department of Animal Medicine, Production and Health, University of Padua, 35020 Legnaro, PD, Italy; barbara.contiero@unipd.it; 5 Institute of Veterinary Physiology, Vetsuisse Faculty, University of Zurich, 8006 Zurich, Switzerland; 6 ASA S.r.l., Via Galileo Galilei 23, 36057 Arcugnano, VI, Italy; davide.travalin@asalaser.com

**Keywords:** photobiomodulation, canine otitis externa, MLS^®^ laser therapy, otic microbiota, otic mycobiota, antimicrobial resistance

## Abstract

This is the first prospective multicenter study investigating photobiomodulation (PBM) in canine OE. Inclusion criteria required an Otitis Index Score 3 (OTIS-3; scale 0–3) ≥1 for erythema and discharge and a Budach cytological score ≥1+ (scale 0–4+) for *Malassezia* spp. and/or bacteria associated with keratinocytes/corneocytes. Twenty-two dogs with OE (38 ears) were enrolled and underwent five PBM sessions. When the discharge score was ≥2, a ceruminolytic ear cleanser was applied before treatment. OTIS-3, Budach cytological scores, and microbiological cultures were evaluated at T0, T2, T4, and T5. Pruritus was assessed using a 10 cm visual analogue scale, and malodour was recorded as present or absent. Early improvement in pruritus and malodour was observed at T2. Median pruritus scores decreased from 5.5 cm (IQR, 4–6) to 2 cm (IQR, 0–4.75), while malodour prevalence declined from 94% to 54%. Cytological evaluation revealed reductions in yeast and keratinocyte scores, whereas microbiological culture results did not significantly change over time. No adverse events were reported. MLS-based PBM was safe and associated with improvement in clinical signs of canine OE.

## Simple Summary

Canine otitis externa (OE) is a common multifactorial inflammatory disease in dogs. Increasing concerns regarding antimicrobial resistance, including the use of topical antimicrobials in recurrent or chronic cases, have stimulated interest in adjunctive non-pharmacological therapy. Photobiomodulation (PBM) is a non-invasive therapy based on the application of near-infrared light that has shown anti-inflammatory, analgesic, and tissue-healing effects in both human and veterinary medicine. The aim of the present prospective multicenter pilot study was to evaluate the feasibility, safety, and preliminary clinical effects of a Multiwave Locked System (MLS^®^)-based PBM protocol in dogs with OE. Twenty-two dogs with OE were treated over five PBM sessions, and clinical signs, cytological findings, and microbiological cultures were evaluated throughout the study period. Improvement was observed in several clinical parameters, while no treatment-related adverse effects were detected. Cytological evaluation also revealed reductions in *Malassezia* yeasts and keratinocytes/corneocytes, whereas microbiological culture results did not show significant quantitative changes over time. These preliminary findings suggest that MLS-based PBM may represent a safe and well-tolerated adjunctive therapeutic option within the multimodal management of canine OE. Given the pilot design and the absence of a control group, these findings should be considered preliminary.

## 1. Introduction

Otitis externa (OE) is an inflammatory condition of the external ear canal that may also involve the pinnae. It is one of the most common dermatological disorders in dogs, with an estimated prevalence of approximately 10% in general veterinary practice [1].

The development and progression of recurrent or chronic OE are multifactorial. The primary, secondary, predisposing, and perpetuating (PSPP) system is commonly used to support diagnosis and treatment planning by systematically identifying the various factors involved in the disease process [2]. Proper identification and management of these components are essential for long-term disease control and for limiting progression to irreversible ear damage, as recurrent inflammation and infection can induce structural and functional changes in the ear canal and reduce therapeutic responsiveness, a process further exacerbated by biofilm formation [3,4].

Topical anti-inflammatory, antifungal, and antibacterial therapies remain the mainstay of treatment for canine OE, while systemic anti-inflammatory medications may be indicated in selected cases [5,6]. Nevertheless, the management of OE continues to be challenging in clinical practice, particularly in recurrent or chronic cases that often require repeated or prolonged treatment courses. In this setting, the long-term or repeated use of antibiotics, even when administered topically, is discouraged due to the increasing concern regarding the development of antimicrobial resistance (AMR) [7,8].

Given these challenges, increasing attention has been directed toward non-pharmacological adjunctive therapies aimed at enhancing treatment efficacy and reducing disease recurrence in canine OE. In this context, a randomized controlled clinical trial evaluating a light-emitting diode (LED)-illuminated gel in dogs with otitis externa reported encouraging clinical outcomes [9]. Although LED-based therapies differ from laser-based approaches, these findings have also contributed to growing interest in photobiomodulation (PBM) within veterinary dermatology [10]. Indeed, PBM is known for its anti-inflammatory and analgesic effects, its ability to reduce edema, and its role in promoting tissue repair and improving microcirculation [11,12].

In parallel, other non-pharmacological approaches have been investigated. Among these, cold atmospheric plasma (CAP), a non-thermal ionized gas that generates reactive oxygen and nitrogen species along with additional physical factors, has been explored as an adjunctive treatment for canine OE [13,14].

Despite the growing interest in PBM and related non-pharmacological strategies for the management of canine OE, no clinical studies have specifically evaluated PBM delivered via a Multiwave Locked System (MLS^®^) laser in this condition. Therefore, the present pilot study was designed to assess the feasibility, safety, and preliminary clinical effects of MLS laser therapy as an adjunctive treatment for canine OE, to evaluate associated cytological and microbiological changes, and to investigate the overall safety, tolerability, and acceptability of this therapeutic approach in client-owned dogs.

## 2. Materials and Methods

### 2.1. Study Design

This study was designed as a multicenter investigation conducted at two referral veterinary hospitals. All procedures were performed in accordance with principles of good clinical practice and with full respect for animal welfare. The study involved client-owned animals and was conducted with informed owner consent. As this was an exploratory pilot study, no formal sample size calculation was performed. The primary aim was to generate preliminary data on the safety, feasibility, and potential clinical effects of MLS-based photobiomodulation in dogs with OE. The data obtained from this pilot study are intended to inform the design, outcome selection, and sample size calculations of future controlled clinical trials.

### 2.2. Eligibility Criteria

Dogs were required to be in good general health based on medical history and physical examination. Diet and the use of nutritional supplements had to remain unchanged for at least three months prior to enrollment and throughout the study period. Routine ectoparasite preventive treatments and topical products, such as shampoos, were permitted, provided they had been unchanged for at least three months before enrollment and were maintained unchanged for the duration of the study. Dogs were excluded if (i) otitis was associated with parasitic infestation, foreign bodies, or neoplasia; (ii) suppurative and/or ulcerative otitis was present; or (iii) rupture of the tympanic membrane was confirmed or suspected. Dogs were also excluded if they had received otic medications or systemic or topical treatment with antibiotics, corticosteroids, non-steroidal anti-inflammatory drugs, or immunomodulatory agents such as JAK inhibitors or cyclosporine within the month preceding enrollment. After application of these criteria, dogs with unilateral or bilateral OE were included if they showed a minimum otitis index score (OTIS-3) [15] of ≥1 for both erythema and discharge, together with a cytological score of ≥1+ according to the Budach scale [16] for *Malassezia* spp. and/or bacteria and keratinocytes and/or corneocytes, based on clinical examination and screening cytology. These thresholds were selected to ensure inclusion of dogs with both clinical and cytological evidence of OE.

### 2.3. Clinical Evaluation

**Figure 1:**
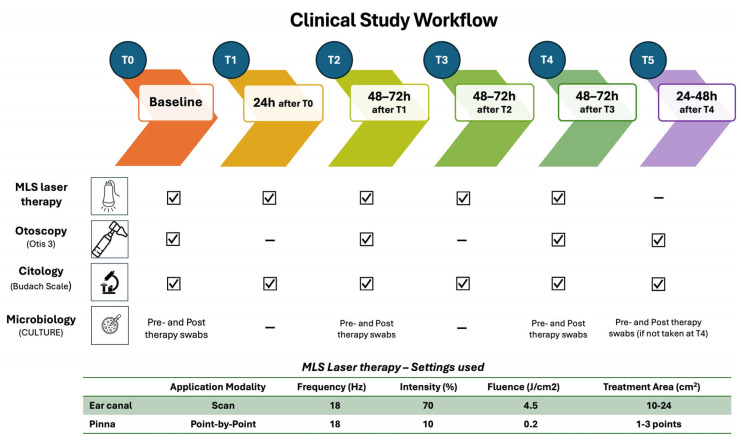
Clinical assessments, cytology, microbiology, and laser therapy performed at each study timepoint.

Clinical evaluations were conducted according to a standardized schedule (Figure 1) at T0, T2, T4, and T5 using the OTIS-3 scoring system, which included assessment of erythema, discharge, swelling, erosion, and ulceration. Each parameter was scored on a scale from 0 (absent) to 3 (severe). The presence or absence of malodour was recorded at each visit. Pruritus was assessed independently at each visit by the dog owner using a visual analogue scale (VAS; 0–10 cm) [17]. A complete otoscopic examination was performed at every visit to evaluate the ear canal and, whenever possible, the integrity of the tympanic membrane. When the pre-treatment discharge score was ≥2, ear cleaning was carried out prior to initiation of the PBM protocol, using a commercially available ceruminolytic ear cleanser containing squalene, salicylic acid, chamomile extract, and tannic acid (Otoact, ICF Srl, Palazzo Pignano, CR, Italy).

### 2.4. PBM Treatment Protocol

All dogs were treated using a Multiwave Locked System (MLS^®^) M-VET Class IV therapeutic laser (ASA Srl, Arcugnano, VI, Italy), which delivers synchronized dual wavelengths: 808 ± 10 nm in continuous and frequency-modulated emission and 905 ± 10 nm in pulsed emission. According to the manufacturer’s technical specifications, the system combines a maximum source power of 4 W for the 808 nm laser and 360 W for the 905 nm pulsed laser. A manufacturer-recommended preset protocol specifically designed for the treatment of canine OE was applied uniformly to all patients. PBM therapy was delivered using two predefined application modalities included in the preset protocol: a scanning mode applied over the external projection of the vertical and horizontal portions of the ear canal and a point-by-point mode applied to the pinna. For treatment of the ear canal, the scanning modality covered standardized areas based on body size category (small < 11 kg, medium 11–22 kg, and large > 22 kg), corresponding to treatment fields of approximately 10 cm^2^, 16 cm^2^, and 24 cm^2^, respectively. Scanning was delivered at a frequency of 18 Hz and 70% intensity, resulting in a standardized dose of 4.5 J/cm^2^ for all size categories. For the pinnae, PBM was delivered using the point-by-point modality. The number of irradiation points depended on body size category: one point in small dogs, two points in medium-sized dogs, and three points in large dogs. Each irradiation point delivered 0.56 J at a frequency of 18 Hz and an intensity of 10% over three seconds, corresponding to a dose of 0.2 J/cm^2^ per point. Based on the treated area and the delivered dose, the total energy delivered per ear per treatment session, distributed across all treated areas (horizontal and vertical ear canal and the pinna when treated), was 45.56 J, 73.12 J, and 109.68 J in small-, medium-, and large-sized dogs, respectively, corresponding to cumulative energies of 227.8 J, 365.6 J, and 548.4 J over the complete five-session treatment protocol. The local fluence remained constant at 4.5 J/cm^2^ for the scanning mode and 0.2 J/cm^2^ per irradiation point for the pinna. These irradiation parameters correspond to those defined in the manufacturer-recommended preset protocol.

The operator wore protective goggles throughout all PBM procedures. Each ear was treated according to a standardized schedule (Figure 1), consisting of five PBM sessions (T0–T4), followed by a final follow-up visit (T5). The second session (T1) was performed 24 h after the baseline session (T0), while subsequent sessions (T2–T4) were conducted at intervals of 48–72 h. The follow-up visit (T5) took place 24–48 h after completion of the final PBM session.

### 2.5. Tolerability

Tolerability of PBM was assessed during and at the end of each treatment session. Treated ears were evaluated by veterinarians for any signs of irritation, including pruritus, pain, or discomfort, as well as for clinical signs of contact dermatitis, such as acute erythema and/or ulceration.

### 2.6. Cytological Examination

Cytological examination was performed for each ear at T0, T2, T4, and T5. Ear swab samples were collected from the junction between the vertical and horizontal portions of the ear canal and stained using a modified Wright’s stain (Diff-Quik; Medion Diagnostics, Düdingen, Switzerland). Slides were prepared at the enrolled veterinary hospitals, while all cytological evaluations were subsequently carried out by a third veterinarian who was blinded to both the clinical data and the PBM treatment schedule. Microscopic examination was performed under oil immersion at ×100 magnification. The cytological parameters evaluated included cocci, rod-shaped bacteria, yeasts, and keratinocytes/corneocytes, which were scored according to the Budach scale. According to this scale, each parameter was graded from 0 to 4 based on the number of elements observed per microscopic field: 0 = none; 1 = rare (detected only after careful examination); 2 = low numbers (readily identifiable); 3 = moderate numbers (easily visible); and 4 = high numbers.

### 2.7. Microbiological Investigation

Microbiological investigations were performed on ear canal swabs collected at predefined study time points and processed at a reference laboratory. As outlined in Figure 1, swabs were obtained at baseline (T0, both before and after treatment) and after selected PBM sessions (T2 and T4), in accordance with the overall study workflow integrating clinical, cytological, and microbiological assessments. At each clinical visit, the attending veterinarian recorded the suspected etiology of OE, selecting one or more among *Malassezia* spp., *Candida* spp., *Pseudomonas* spp., and the *Staphylococcus pseudintermedius* group (SIG), representing microorganisms of clinical relevance in canine OE [18].

These clinical suspicions guided the choice of targeted laboratory analyses. Accordingly, not every swab underwent all microbiological analyses, as only the laboratory investigations corresponding to the clinically suspected pathogen(s) were performed.

All microbiological analyses were conducted by laboratory personnel blinded to the clinical data, treatment allocation, and sampling time points. Each swab was suspended in buffered peptone solution and plated onto selective agar media appropriate for the suspected pathogens. Plates were incubated at 37 °C for 48 h for bacterial isolates and *Candida* spp. and for up to 5 days for *Malassezia* spp., with interim evaluations performed at 48–72 h. Colony counts were performed manually. In cases of heavy bacterial growth, particularly for SIG isolates, plates were subdivided into halves or quadrants; colonies were counted in one section and extrapolated to the total plate area. For each sample, colony counts from the last two dilutions showing growth were totaled, and the final quantitative value was calculated according to the standardized method described in ISO 7218:2007/Amd 1:2013 [19], which takes into account the dilution factor and the volume plated in standard plate count enumeration. Preliminary identification of isolates was based on colony morphology and subsequently confirmed with MALDI TOF mass spectrometry. In addition, isolates belonging to the SIG were further characterized by endpoint multiplex PCR to differentiate *Staphylococcus pseudintermedius* from closely related species.

### 2.8. Statistical Analysis

All statistical analyses were performed using methods appropriate for ordinal or non-normally distributed data. Clinical, cytological, and visual analogue scale (VAS) scores (0–10 cm) for pruritus were analyzed using generalized linear mixed models (GLMMs) for ordinal multinomial outcomes with a cumulative logit link function. Time was included as a fixed effect, while dog was included as a random intercept to account for the correlation between repeated observations from the same individual, including dogs contributing bilateral ears. Pairwise comparisons among study time points were performed. The binary presence or absence of malodour was assessed with Cochran’s Q test for paired proportions. Microbiological outcomes, expressed as log10 colony-forming units (CFUs) per swab, were analyzed using Kruskal–Wallis tests because not all microbiological samples were available at each time point, and pathogen-specific datasets differed in sample size according to the predefined microbiological workflow, making repeated-measures modeling inappropriate for these outcomes. The relationship among cytological findings (cocci, rods, yeasts, keratinocytes, and corneocytes) and microbiological culture results was investigated using Spearman’s rank correlation coefficient. Significance was set at *p* < 0.05. Analyses were performed using commercial SAS software (Version 9.4; SAS Institute Inc., Cary, NC, USA) and XLSTAT (Version 2025.2.1; Addinsoft, Paris, France).

## 3. Results

### 3.1. Study Population

Twenty-two dogs were enrolled in the study, accounting for a total of 38 ears affected by OE. Otitis was predominantly bilateral; however, some dogs presented with unilateral disease. The study population had a median age of 6.5 years (IQR, 4–10) and a median body weight of 28.8 kg (IQR, 22–35). Sixteen males (fourteen intact and two neutered) and six spayed females were included. Based on body size, dogs were classified as small (n = 1), medium (n = 10), or large (n = 11). Mixed-breed dogs comprised 36% of the study population (n = 8). The remaining dogs belonged to the following pure breeds: Labrador Retriever (n = 3), Boxer (n = 1), Pointer (n = 1), Maremmano Abruzzese Sheepdog (n = 1), Maltese (n = 1), Weimaraner (n = 1), Cane Corso (n = 1), German Shepherd (n = 1), Golden Retriever (n = 1), Bullmastiff (n = 1), Basset Hound (n = 1), and Cocker Spaniel (n = 1). Among enrolled dogs, 7 of 22 were diagnosed with atopic dermatitis as the primary underlying cause of otitis externa. In the remaining cases, a definitive primary cause was not identified. Two dogs discontinued participation after T2 due to the owners’ perception of insufficient early clinical improvement. Discontinuation was not associated with treatment tolerability issues or the occurrence of adverse effects. No pain, behavioral changes, or treatment-related adverse events were reported by owners at any study visit.

### 3.2. Clinical Outcome

**Figure 2:**
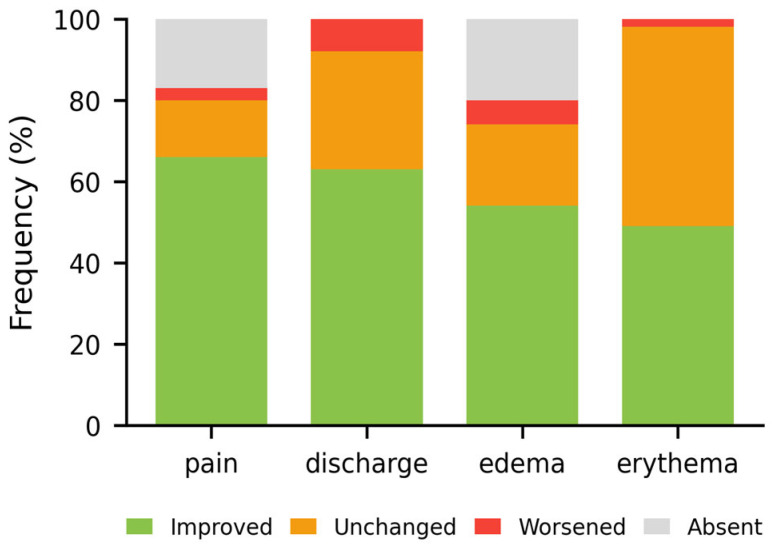
Percentage of ears (n = 38) showing improvement, unchanged clinical signs, worsening, or absence of each clinical sign over the study period. Improvement was defined as a decrease in score, unchanged as no variation in score, worsening as an increase in score, and absence as a score of 0 (clinical sign not present).

**Figure 3:**
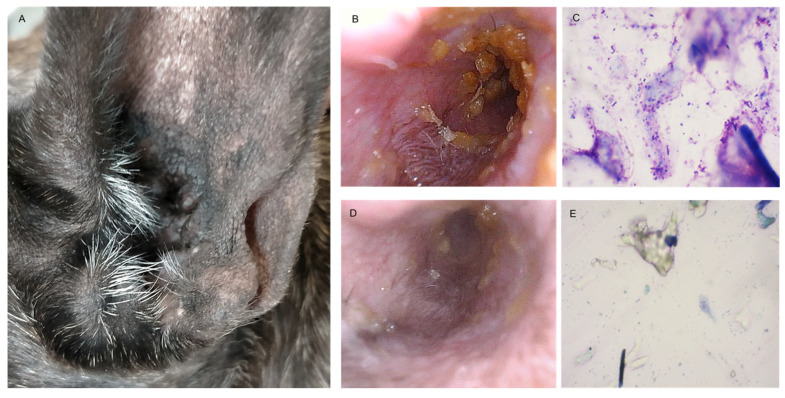
Representative images from the right ear of a dog enrolled in the study: (**A**) Clinical appearance of the pinna at baseline (T0). (**B**) Video-otoscopic view of the external ear canal at T0, showing mild erythema and ceruminous discharge. (**C**) Representative cytological image at T0, showing numerous *Malassezia* yeasts. (**D**) Video-otoscopic view of the external ear canal at the end of treatment (T5), showing reduced ceruminous discharge. (**E**) Representative cytological image at T5, showing marked reduction in *Malassezia* yeasts.

A statistically significant improvement was observed in most clinical parameters over the study period (Table 1). Median erythema scores decreased from 2 (interquartile range [IQR], 1.5–3) at T0 to 1 (IQR, 1–2) at T5 (*p* < 0.001). A significant reduction in edema was detected over time, with improvement already evident at T2 and maintained through T5 (Table 1). Discharge and pain scores also decreased significantly, with clinically relevant improvement observed from T4 onward. Pruritus showed a consistent reduction from a median of 5.5 cm (IQR, 4–6) at T0 to 2 cm (IQR, 0–4.75) at T5 (*p* < 0.001). Malodour, which was present in 94% of ears at baseline, decreased to 54% by the end of the treatment protocol (*p* < 0.001), with a significant reduction already observed at T2. In contrast, erosion scores remained unchanged throughout the study period (*p* > 0.05), likely due to low baseline values and the limited number of affected ears (n = 3). Correlation analysis showed that, at T5, ears with higher pruritus scores were more likely to present persistent erythema, edema, and discharge, indicating an incomplete clinical response in a subset of cases. As illustrated in Figure 2, most ears showed improvement between T0 and T5 for pain, discharge, edema, erythema, and pruritus. Representative clinical images of the external ear canal before and after treatment are shown in Figure 3.

### 3.3. Cytology

**Figure 4:**
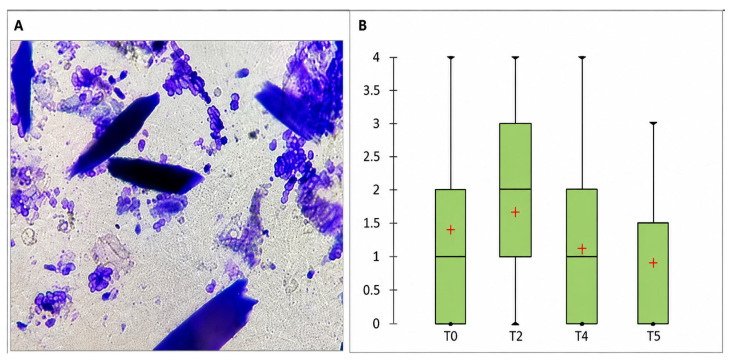
(**A**) Representative ear cytology (100× oil immersion) showing numerous *Malassezia* yeasts associated with corneocytes. (**B**) Box-and-whisker plot of the *Malassezia* cytological scores at the different time points. The red + indicates the mean, the black horizontal line the median, and the box represents the interquartile range (25th–75th percentiles). Whiskers indicate the data range.

**Figure 5:**
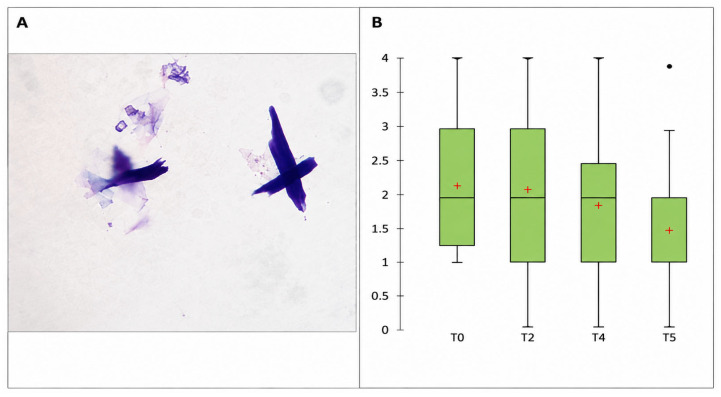
(**A**) Representative ear cytology (100× oil immersion) showing corneocytes. (**B**) Box-and-whisker plot of the keratinocyte scores at different time points. Red + is the mean, the black line corresponds to the median, and the bottom and top of the box represent the first and the third quartiles, respectively. Whiskers correspond to the interquartile range. Dots are outliers.

Cytological findings revealed selective and time-dependent improvements. Yeast scores decreased significantly from a median of 1 (0–2) at T0 to 0 (0–1.5) at T5 (*p* = 0.001) (Figure 4). A similar dynamic was observed for keratinocytes/corneocytes, which declined from 2 (1.3–3) to 1 (1–2) (*p* < 0.001) (Figure 5). In contrast, cocci and rods showed no significant variation over time (*p* = 0.082 and 0.432, respectively).

### 3.4. Microbiology

Quantitative culture analysis did not reveal significant changes in microbial load over time for any of the investigated pathogens. For the SIG, positive cultures were identified in 12 ears at baseline (T0), in 12 immediately post-treatment at T0, in 10 at T2, and in 8 at T4. No relapses were recorded during the study period. The median duration of culture positivity was 6 days (IQR, 3–9 days). *Pseudomonas aeruginosa* exhibited greater temporal variability, with positive cultures detected in 5 ears at T0, in 6 immediately post-treatment at T0, in 7 at T2, and in 5 at T4. The median duration of positivity for *Pseudomonas* spp. was 4 days (IQR, 1–7 days), and no relapses were observed. *Malassezia* spp. were cultured from 11 ears at T0, from 10 immediately post-treatment at T0, from 7 at T2, and from 10 at T4. Both persistent and newly positive samples were observed across time points, with five documented recurrences. The median duration of culture positivity was 4 days (IQR, 1–7 days). *Candida* spp. were infrequently isolated, with positive cultures detected in 3 ears at T0, in 1 immediately post-treatment at T0, in one at T2, and in 1 at T4. Owing to the small number of positive samples, inferential statistical analyses were not performed, and only descriptive statistics are reported. The mean duration of positivity was 3 days (standard deviation, 4 days). A statistically significant positive correlation was observed between yeast cytological scores and quantitative SIG culture results at T2 (r = 0.665; *p* = 0.042).

### 3.5. Limitations

The limited sample size may have reduced the statistical power to detect differences in some outcome measures, particularly microbiological outcomes and clinical signs with low baseline prevalence. Therefore, non-significant results should not be interpreted as evidence of absence of effect, and the findings should be considered preliminary and hypothesis-generating.

## 4. Discussion

To the authors’ knowledge, this study represents the first prospective multicenter investigation evaluating PBM delivered using an MLS laser system for the management of canine OE. PBM is a non-thermal therapeutic modality based on the application of red and near-infrared (NIR) light, typically within a wavelength range of approximately 600–1100 nm [20]. Although PBM has been extensively investigated, its mechanisms of action are only partially understood. Current hypotheses suggest that light absorption by intracellular chromophores, particularly mitochondrial components such as cytochrome c oxidase, as well as potentially by light-sensitive ion channels, may modulate cellular metabolism and activate downstream signaling pathways [21,22]. Together with the regulation of reactive oxygen species and nitric oxide-mediated pathways, these effects are thought to influence inflammation, cellular proliferation, and tissue repair [23]. In addition, PBM may modulate pruritus through its effects on neural and nociceptive pathways activated by inflammatory mediators at the level of peripheral sensory nerves [24,25]. Finally, biological responses to PBM appear to depend on multiple treatment parameters, among which the delivered energy dose (fluence) is a key determinant of therapeutic efficacy and clinical outcome [26]. In this study, dogs affected by OE showed significant clinical improvement across several parameters, including erythema, edema, discharge, pain, pruritus, and malodour. Overall, these findings were consistent with a potential anti-inflammatory effect associated with PBM. Supporting this interpretation, experimental evidence from animal models of OE suggests that exposure to red and near-infrared light may reduce inflammatory burden and biofilm-related pathology in otic tissues [27]. These findings further support the hypothesis that the clinical benefits observed in the present study may be primarily related to modulation of the host inflammatory response rather than to a direct antimicrobial effect.

Accordingly, the reduction in erythema and edema observed in this study may have reflected a progressive resolution of inflammation, which is known to drive epithelial hyperplasia and increased glandular activity that ultimately leads to excessive cerumen production [28].

In parallel, the decrease in discharge may have been driven by normalization of epithelial turnover and glandular secretion, together with restoration of epithelial migration within the ear canal. Consequently, the observed improvement in pain, pruritus, and malodour may have reflected effective control of inflammation and irritation, as these signs represent characteristic clinical manifestations of canine OE and are directly linked to inflammatory processes that negatively affect the quality of life of both dogs and their owners [29]. Cytological findings further supported this interpretation, as a significant reduction in keratinocytes/corneocytes and *Malassezia* spp. overgrowth was also observed. The decrease in keratinocytes/corneocytes may, at least in part, have reflected normalization of epithelial turnover and a reduction in surface inflammation [21,23]. These changes may have contributed to restoration of the local ear canal microenvironment, including epithelial integrity, cerumen production, and lipid composition [30].

*Malassezia* spp. are lipid-dependent yeasts whose proliferation is closely associated with increased cerumen production, altered lipid composition, and inflammatory changes within the ear canal. Previous studies have reported that clinical improvement in canine OE may be accompanied by a reduction in *Malassezia* spp., likely secondary to modulation of the local ear canal environment [31,32]. Alongside these findings, there was a lack of significant changes in bacterial counts based on cytology. Consequently, the marked clinical improvement observed in the present study despite the absence of significant microbiological changes may reflect restoration of the ear canal microenvironment, including epithelial integrity, cerumen composition, and epithelial homeostasis. However, because this pilot study did not include a control, these findings should be interpreted as preliminary and hypothesis-generating.

Experimental data suggest that light irradiation may influence microbial behavior and bacterial growth dynamics. However, the reported effects appear to be highly dependent on several factors, including wavelength, delivered energy dose, microbial species, and experimental conditions [33]. Microbiological cultures failed to demonstrate statistically significant changes in either yeast or bacterial isolates. This apparent discrepancy between cytological and microbiological findings is not unexpected and has been previously reported in canine OE, where only partial agreement between diagnostic methods has been observed [34]. Cytology reflects the microorganisms directly visualized within the sampled ear canal and provides information on their abundance and inflammatory response, whereas culture detects only cultivable organisms and may underestimate the complexity of the microbial community [35,36,37]. Studies employing high-throughput sequencing have shown that the microbial composition of affected canine ears is primarily characterized by reduced microbial diversity and shifts in community structure, rather than by simple quantitative overgrowth of individual microorganisms. Consistently, investigations comparing culture-based and molecular approaches for characterization of the canine ear microbiome have demonstrated that conventional culture methods may underestimate microbial diversity and may fail to capture the full complexity of microbial communities. In this context, it has been suggested that PBM may influence microbial ecosystems indirectly through modulation of the local biological environment [38,39]. These limitations also apply to fungal populations, including *Malassezia* spp., whose composition within the otic mycobiota may not be fully captured by standard culture-based methods [40,41,42]. Therefore, the absence of statistically significant changes in culture results should be interpreted with caution, as culture-based methods may not fully capture biologically relevant modifications of the otic microbiota and mycobiota. Longer observation periods and the inclusion of molecular approaches would be useful to better clarify whether PBM contributes to modulation of the otic microbial and fungal ecosystems beyond what is detected using conventional culture techniques.

An important finding of this study is the safety and tolerability profile of PBM. No adverse events or treatment-related pain were observed during the treatment sessions. These results are consistent with previous reports in veterinary medicine, in which PBM has been described as a safe and non-invasive therapeutic modality across a wide range of clinical indications [43,44].

Some limitations of this study should be acknowledged. The relatively small sample size may have reduced the statistical power to detect differences for some outcome measures, and the findings should therefore be interpreted as preliminary. The relatively short follow-up period also precluded evaluation of disease recurrence, a clinically relevant aspect of canine OE. In addition, because the study population included dogs with different underlying clinical conditions, including atopic dermatitis in seven dogs, the present study was not designed to determine whether clinical response or recurrence differed according to the underlying etiology.

Another potential limitation could have been the use of a ceruminolytic ear cleaner in ears with pre-treatment discharge scores ≥2, which may have influenced clinical and cytological findings. Ear cleaning was performed only when clinically indicated by pre-treatment discharge scores ≥2, consistent with the routine management of canine OE. Although ear cleaning is considered a standard component of canine OE management and contributes to the removal of cerumen and debris, previous studies have generally evaluated repeated cleaning protocols or ear cleaning combined with topical therapy [35,45], whereas in the present study only a single application of a ceruminolytic agent was performed. Furthermore, recent evidence suggests that ear cleaning alone has a limited impact on short-term treatment outcomes in most cases of canine OE [46]. Therefore, in the absence of a control group, it was not possible to distinguish the effects of PBM from spontaneous clinical improvement or from the potential contribution of ceruminolytic ear cleaning performed in a subset of dogs.

Finally, reliance on culture-based microbiological methods may have restricted the detection of changes in microbial community structure.

## 5. Conclusions

In conclusion, this prospective multicenter pilot study suggests that MLS-based PBM was safe and well tolerated in dogs with OE. The observed findings are consistent with potential modulation of inflammation and restoration of the ear canal microenvironment. These encouraging results support further evaluation of MLS-based PBM as a potential adjunctive therapeutic approach for canine OE. However, given the pilot nature of the study, the absence of a control group, and the limited follow-up period, the findings should be interpreted with caution. Consequently, although clinical improvement was observed during the study period, a causal therapeutic effect of PBM cannot be established from the present data.

Future controlled clinical studies with longer follow-up periods, together with the integration of molecular approaches to characterize changes in the otic microbiota and mycobiota, are warranted to further define the role of PBM in the management of canine OE.

## Figures and Tables

**Table 1:** Clinical scores over time in dogs with otitis externa treated with MLS-based PBM therapy. Different superscript letters along rows mean significantly different values (*p* < 0.05).

	Ears (n)	T0	T2	T4	T5	*p*
Erythema (0–3)	35	2 (1.5–3) ^a^	1 (1–2) ^b^	2 (1–2) ^b^	1 (1–2) ^b^	<0.001
Edema (0–3)	35	1 (0.5–2) ^a^	1 (0–1) ^b^	1 (0–1) ^b^	0 (0–1) ^b^	<0.001
Erosion (0–3)	35	0 (0–0)	0 (0–0)	0 (0–0)	0 (0–0)	0.707
Discharge (0–3)	35	2 (1–3) ^a^	1 (1–2) ^b^	1 (1–2) ^bc^	1 (0–1.5) ^c^	<0.001
Pain (0–3)	35	1 (1–2) ^a^	1 (0–2) ^b^	0 (0–1) ^c^	0 (0–1) ^c^	<0.001
Malodour (% of affected ears)	35	94% ^a^	63% ^b^	54% ^b^	54% ^b^	<0.001
Pruritus VAS (1–10 cm)	30	5.5 (4–6) ^a^	4 (3–6) ^b^	3 (1–4.75) ^c^	2 (0–4.75) ^c^	<0.001

## Data Availability

The data presented in this study are available from the corresponding author upon reasonable request.

## Abbreviations

The following abbreviations are used in this manuscript:

AMR	Antimicrobial resistance
CAP	Cold atmospheric plasma
CFU	Colony-forming units
IQR	Interquartile range
LED	Light-emitting diode
LLLT	Low-level laser therapy
MLS	Multiwave Locked System
NIR	Near-infrared
OE	Otitis externa
OTIS-3	Otitis Index Score 3
PBM	Photobiomodulation
PSPP	Primary, secondary, predisposing, and perpetuating
SIG	*Staphylococcus pseudintermedius* group
VAS	Visual analogue scale
